# Impact of Career Design Simulation on Japanese Medical Students: An Educational Study

**DOI:** 10.7759/cureus.65382

**Published:** 2024-07-25

**Authors:** Nobuyasu Komasawa, Masanao Yokohira

**Affiliations:** 1 Community Medicine Education Promotion Office, Faculty of Medicine, Kagawa University, Miki-cho, JPN; 2 Department of Medical Education, Kagawa University, Miki-cho, JPN

**Keywords:** doctorate degree, specialty board, simulation, medical student, career designing

## Abstract

Background: The present study aimed to evaluate the effect of career design simulation on the attitude of Japanese medical students toward doctoral life planning, including qualification acquisition.

Methods: In this study, we conducted a career-design simulation trial with fourth-year medical students. The three-hour course comprised lectures on career design and individual career design, including an ideal plan and a modified plan according to unexpected work and life events, as well as group discussions. Nine questions regarding career design attitude (awareness, significance, or intention to acquire a doctoral degree or medical specialty, work-life balance, and flexibility) were answered in both pre- and post-career design simulation training.

Results: The response rate was 67.5% (79/117). Attitudes toward career design significantly improved after the career design simulation, regardless of the duration after graduation (p < 0.001, each). Attitudes toward the significance of doctorate degrees significantly improved after participation (p = 0.007), while attitudes toward medical specialties did not (p = 0.128). In contrast, confidence in obtaining a medical specialty and doctoral degree was improved by career design simulations (p = 0.009 and p = 0.003, respectively).

Conclusion: These findings suggest that career design simulations can be an effective educational method for developing active learning, motivation for career image, and lifelong education.

## Introduction

In Japan, students have the option to enter medical school directly after completing high school at the age of 18, provided they have not repeated any years or taken a preparatory period for university entrance [1,2]. Upon completing medical school and acquiring a medical doctor’s degree (M.D.) by passing the national examination, most students undergo a mandatory two-year clinical training (initial training) period, typically at ages 24-26. Subsequently, most specialize in medical fields such as internal medicine, general surgery, pediatrics, or critical care, which usually entail an additional 4-5 years of training. After achieving specialty board certification (approximately 7-10 years after graduation from medical school), they embark on a lifelong learning journey to enhance their skills and performance [3]. Japanese doctors are encouraged to pursue doctoral degrees once they have gained research experience. Consequently, young doctors encounter various complex steps and decisions at the outset of their careers [4]. The decline in the number of medical doctors obtaining doctoral degrees presents a significant issue in Japan, as this trend is closely linked to a deterioration in research capabilities within the medical field [5].

Previously, we discussed the importance of career planning awareness among Japanese medical students and highlighted that their understanding of career design, particularly lifelong planning, was generally unclear [6]. We revealed that although medical students display acute awareness during the initial training period after graduation, their attention toward future events, such as medical specialty training and lifelong learning, appears to be less defined. Furthermore, the aspiration to pursue a doctoral degree appears notably weaker than that for a medical specialty. Our study revealed a clear need for systematic and longitudinal career development programs tailored to medical students.

To address this critical situation, structured career design education and training are essential to allow medical students to develop skills for lifelong career planning and adaptability to unforeseen work and life circumstances. As simulation-based education encourages active learning and aids long-term retention [7,8], we created a career design simulation for educational purposes and assessed its primary educational impacts in this study.

In this study, we used a simulation-based career design for fourth-year medical students at our university and evaluated its educational effects.

## Materials and methods

Ethical considerations

This study was approved by the Research Ethics Committee of the Faculty of Medicine at Kagawa University (No. 2023-024). Verbal informed consent was obtained from students by medical teachers, and clerks witnessed the process before the survey. All students were informed of the nature and purpose of the study, and their anonymity was guaranteed. Students were also informed that they had the opportunity to withdraw from the study within a week after they responded to the survey. We also emphasized that withdrawing from the study would not influence their academic outcomes in any way. This study population included no minors, as all fourth-year medical students in Japan are > 21 years old [9].

Setting

The participants were recruited from the Faculty of Medicine at Kagawa University, the only medical school in Kagawa Prefecture. Kagawa Prefecture is located northeast of the Shikoku region. Kagawa Prefecture is located northeast of the Shikoku region and has a population of 960,000 people.

Career design simulation

The career design simulation was performed on April 10, 2024, in the class “Medical management and diagnosis,” in which fourth-year medical students learn basic clinical approaches and lifelong learning. The simulation lasted 3 hours.

In the first hour, the medical teacher gave lectures on career design basics, including qualification acquisition, such as a specialty board or doctoral degree.

In the middle of the hour, medical students performed career design simulations on their own. Medical students wrote about their own career designs. The career design simulation rules are presented in Table 1.

**Table 1:** Rule of career design simulation

Rules
Specialty board training span is four years
Graduate school for obtaining doctorate degree (Ph.D.) is four years
Research period for obtaining doctorate degree (Ph.D.) without graduate school is seven years
Add period (e.g., XX years) for each major event
There is no constant retirement point
Construct Plan A is ideal plan, Plan B modified plan due to unexpected factor associated with work, and Plan C modified form due to unexpected factor associated with life

**Figure 1:**
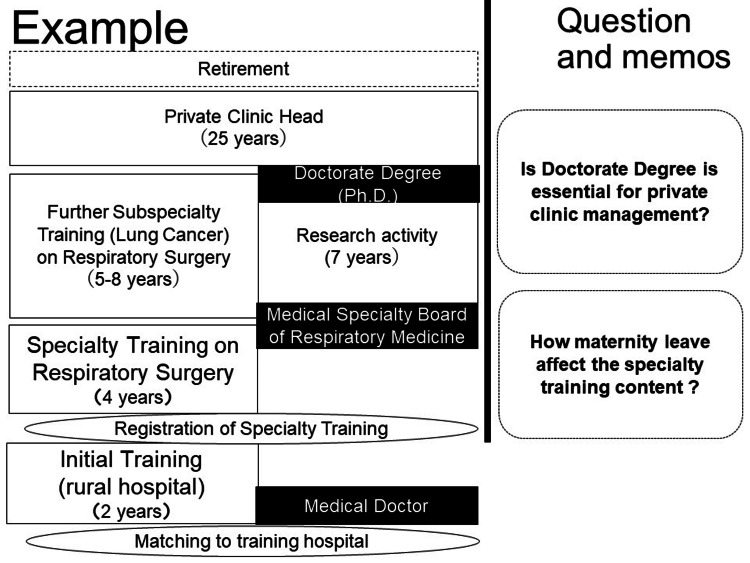
Career design simulation example was used in this study

They created Plan A, an ideal plan; Plan B, a modified plan due to unexpected factors associated with work; and Plan C, a modified plan due to unexpected factors associated with life. Examples of ideal types are depicted in Figure 1.

In the last hour, they discussed their career designs with each other in small groups of approximately 6-7 students. Medical teachers provided feedback on students’ questions.

Study measures and population

We conducted a questionnaire survey to evaluate the educational effects of career design simulations, both pre- and post-career design simulations. The same questions were answered in both the pre- and post-questionnaires. The contents reveal their awareness of career design after graduation and their intention to acquire a medical specialty board and obtain a doctoral degree. The questionnaire contents are listed in Table 2.

**Table 2:** Questionnaire toward medical students pre- and post-career design simulation. The same question was performed for evaluating the educational effect

	Questions
Q1	Are you confident in your career design for 1-2 years after graduation?
Q2	Do you have confidence in your career design for 3-7 years after graduation?
Q3	Do you have confidence in your career design from 8 years after graduation?
Q4	Do you believe there is significance in obtaining a Doctorate Degree?
Q5	Do you believe there is significance in obtaining a Medical Specialty board?
Q6	Do you have confidence in smoothly obtaining a Doctorate Degree?
Q7	Do you have confidence in smoothly obtaining a Medical Specialty board?
Q8	Do you have the confidence to maintain a work-life balance while building your career?
Q9	Do you think you can adapt flexibly to future career changes and uncertainties?(Flexibility toward career design)

The themes were generated by referring to several review articles and previous questionnaire studies [6], and responses were rated on a five-point Likert scale (5 = strongly agree to 1 = strongly disagree) [10]. Q1-Q3 are evaluations of career design confidence from the viewpoint of time flow. Q4-Q7 is an evaluation of their subjective significance and confidence in acquiring a doctoral degree or a medical specialty board. Q8 is confidence toward the maintenance of work-life balance, while Q9 is flexibility in career design. The contents of the questionnaire were evaluated by three medical education professionals. A pilot test was then performed by four medical clerks from our center.

Study population

Japanese medical schools usually have a six-year study period. Students can enter medical school after graduating from high school and successfully passing an entrance exam. As with other medical schools in Japan, medical students at the Faculty of Medicine at Kagawa University complete all basic and clinical medicine lectures and skills training before beginning a clinical clerkship, typically in the fourth grade [11,12]. This career design simulation was performed during the preparation course before beginning clinical clerkship, and participants did not undergo any systematic career design education.

Patient and public involvement

There was no patient or public involvement.

Statistical analysis

Statistical analyses were performed using the JMP Pro version 13.2.1 software (SAS Institute Inc., Cary, NC, USA). A chi-square test was used to compare the pre- and post-career design simulations [13]. Data are presented as mean ± standard deviation. P-values < 0.05 were considered statistically significant.

## Results

In total, 79 of 117 fourth-year medical students responded to the survey (response rate: 67.5%). A comparison of medical students’ attitudes toward career design between pre- and post-career design simulations is presented in Table 3.

**Table 3:** Comparison of attitude toward career design between pre- and post-career design simulations in medical students (Likert scale: strongly agree, agree, somewhat agree, disagree, strongly disagree) The number of respondents to each Likert scale option is shown. *p<0.05 was considered significant by the Chi-square test.

	Pre					Post					P-value
	Strongly agree	Agree	Somewhat agree	Disagree	Strongly disagree	Strongly agree	Agree	Somewhat agree	Disagree	Strongly disagree	
Q1	1	1	14	55	8	4	12	36	27	0	<0.001*
Q2	0	0	8	60	11	2	9	32	35	1	<0.001*
Q3	0	2	7	58	12	3	7	29	39	1	<0.001*
Q4	6	10	56	7	0	12	26	37	4	0	0.007*
Q5	18	29	31	1	0	30	31	17	1	0	0.128
Q6	1	3	6	61	8	4	9	15	49	2	0.009*
Q7	1	6	31	40	1	5	17	37	20	0	0.003*
Q8	0	4	23	48	4	4	11	27	33	4	0.051
Q9	0	7	21	49	2	3	9	40	26	1	0.049*

Career design attitude significantly improved after career design simulation, regardless of the duration after graduation (Q1-Q3, p < 0.001, each). Attitudes toward the significance of a doctoral degree significantly improved after participation (p = 0.007), while attitudes toward medical specialties did not (p = 0.128). In contrast, confidence in obtaining a medical specialty or doctoral degree was improved by career design simulations (p = 0.009 and p = 0.003, respectively). The attitude toward the maintenance of work-life balance did not show a significant difference after the simulation (p = 0.051).

## Discussion

While most Japanese medical practitioners opt for a clinical career path, some choose not to engage in clinical practice [14]. Valuable contributions to enhancing patient outcomes have indeed originated from medical professionals in non-clinical domains such as policy or research. Although most medical students in Japan initially focus on clinical specialties, a subset may later explore non-clinical domains, such as basic research and social medicine. A foundation in medicine and clinical experience is beneficial for pursuing alternative paths [15]. Consequently, prudent backup planning in designing a medical career is crucial, particularly when confronted with the potential inability to achieve the primary career objective [16]. From this viewpoint, career design simulation, which cultivates doctoral life planning, is warranted in the present medical education framework.

This study evaluates the educational effects of a career design simulation on fourth-year medical students. This educational intervention significantly improved the medical students’ attitudes toward career design after graduation. As recognition of the significance of the medical specialty did not significantly differ before and after the simulation, this tendency can be associated with the fact that medical students displayed a strong attitude toward the medical specialty even before the simulation. Notably, the significant improvement in career design flexibility indicated the possibility that career design simulations could enhance management skills toward career development in medical students. This also suggests that simulation-based education in doctoral life design can enhance career development skills. In contrast, the attitude toward work-life balance did not improve significantly by career design simulation. This suggests that basic knowledge accumulation on work-life balance or burnout is essential, and further curriculum evaluation is warranted.

Advancements in medicine have heightened expectations of the knowledge and skills required by medical doctors [17]. The education provided by medical schools alone falls short of meeting this demand. Therefore, medical practitioners must continuously shape their careers and refine their knowledge and skills throughout their professional lives [18]. Commencing career design during the initial clinical training period after obtaining an M.D. may be too late to manage the complex tasks expected of medical students during their careers. Early exposure to clinical settings and guided research experiences can positively affect medical students’ ability to design their careers and commit to lifelong education [19]. Career counseling during medical school represents another potential source of support.

Career guidance programs, workshops, and specialty student interest groups can provide advice to medical students and young doctors [20]. Thus, incorporating career design education, including research skill acquisition and enhancing the understanding of the value of a doctoral degree alongside clinical practice, can be effective for medical students.

Simulation training is a highly effective instructional tool that benefits both technical and non-technical behavior-based crisis management skills [21]. Effective clinical outcomes and advancements in patient safety during the perioperative period necessitate both technical and non-technical skills. Simulation experiences allow practitioners to practice and maintain rarely used resuscitation or rapid response skills, which are categorized as non-technical skills [22]. These skills encompass the cognitive, social, and personal resources that complement technical abilities and contribute to safe and efficient task performance. Non-technical skills enhance technical proficiency and usually encompass teamwork skills in domains like situation awareness, decision-making, communication, teamwork, leadership, and stress and fatigue management [23]. The inclusion of non-technical skills training is a standard practice in patient or medical safety training programs. Deficiencies in these skills may increase the risk of errors, thereby increasing the likelihood of adverse events or clinically relevant adverse outcomes. Given that one of the primary goals of career design is to acquire adaptable skills [24,25], career design simulations can facilitate the development of non-technical skills necessary for adjusting or managing one’s career over time.

This study has several notable limitations that need to be acknowledged. First, it is important to recognize that the data were collected only from one institution. Therefore, caution must be exercised when attempting to generalize our findings to other medical schools. However, because the Model Core Curriculum for Medical Education is widely used in all institutions, our findings probably apply to medical schools across Japan. The Model Core Curriculum for Medical Education serves as a structured model outlining the essential components universally addressed by all Japanese universities when developing their respective medical education curricula [26]. Moving forward, conducting a nationwide survey on attitudes toward career development among Japanese medical students would be beneficial. Second, the analysis included participants of both sexes. Exploring gender differences in career design simulations is an intriguing avenue for further investigation [27]. Finally, our quantitative analysis of career design relied on a visual analog and a Likert scale to assess awareness of and attitudes toward career design. Future research should consider incorporating additional quantitative analyses, such as interviews or text-mining analyses, into portfolios following career design simulation [28,29].

In the future, it may be significant to expand career design simulations to include other medical students, such as those studying nursing or pharmacology, as well as individuals in residency and throughout their lifelong education journey [30]. Furthermore, the effect of career design simulation on academic achievement is also significant.

## Conclusions

We performed a career design simulation for medical students at our university and examined the educational effects. Our results suggest that career design simulations for medical students may be effective in promoting active learning and cultivating skills related to career development and lifelong learning.

## References

[1] Yoshii I, Sawada N, Chijiwa T (2023). Clinical significance of serum cystatin C-to-creatinine ratio as a surrogate marker for incident osteoporotic fracture predictions. J Gen Fam Med.

[2] Komasawa N, Terasaki F, Takitani K, Lee SW, Kawata R, Nakano T (2022). Comparison of Younger and Older medical student performance outcomes: A retrospective analysis in Japan. Medicine (Baltimore).

[3] Komasawa N, Terasaki F, Kawata R, Nakano T (2022). Gender differences in repeat-year experience, clinical clerkship performance, and related examinations in Japanese medical students. Medicine (Baltimore).

[4] Park YS, Hicks PJ, Carraccio C, Margolis M, Schwartz A (2018). Does incorporating a measure of clinical workload improve workplace-based assessment scores? Insights for measurement precision and longitudinal score growth from ten pediatrics residency programs. Acad Med.

[5] Shibayama S, Kobayashi Y (2017). Impact of Ph.D. training: a comprehensive analysis based on a Japanese national doctoral survey. Scientometrics.

[6] Komasawa N, Yokohira M (2024). Attitude toward career development in Japanese medical students: a questionnaire survey. BMJ Open.

[7] Cheng A, Belanger C, Wan B, Davidson J, Lin Y (2017). Effect of emergency department mattress compressibility on chest compression depth using a standardized cardiopulmonary resuscitation board, a slider transfer board, and a flat spine board: a simulation-based study. Simul Healthc.

[8] Issenberg SB, McGaghie WC, Petrusa ER, Lee Gordon D, Scalese RJ (2005). Features and uses of high-fidelity medical simulations that lead to effective learning: a BEME systematic review. Med Teach.

[9] Komasawa N, Yokohira M (2024). Comparison of attitudes toward community-based medicine between regional-quota and general-selected medical student in Japan. J Rural Med.

[10] Komasawa N, Fujiwara S, Atagi K (2014). Effects of a simulation-based sedation training course on non-anesthesiologists' attitudes toward sedation and analgesia. J Anesth.

[11] Komasawa N, Terasaki F, Nakano T, Kawata R (2020). Relationships between objective structured clinical examination, computer-based testing, and clinical clerkship performance in Japanese medical students. PLoS One.

[12] Komasawa N, Terasaki F, Nakano T, Kawata R (2021). Correlation of student performance on clerkship with quality of medical chart documentation in a simulation setting. PLoS One.

[13] Komasawa N, Berg BW, Minami T (2018). Problem-based learning for anesthesia resident operating room crisis management training. PLoS One.

[14] Durning SJ, Artino AR Jr, Pangaro LN, van der Vleuten C, Schuwirth L (2010). Perspective: redefining context in the clinical encounter: implications for research and training in medical education. Acad Med.

[15] Pols DH, Kamps A, Runhaar J, Elshout G, van Halewijn KF, Bindels PJ, Stegers-Jager KM (2023). Medical students' perception of general practice: a cross-sectional survey. BMC Med Educ.

[16] Wahlberg K, Mughal A, Li Z, Cipolla MJ, Cushman M, Flyer JN (2022). Retrospective study of medical student scholarship and career trajectory following a mentored preclinical cardiovascular summer research fellowship. BMJ Open.

[17] Conyers L, Tackett S, Wright S (2022). Internal medicine-paediatrics residents' application of life design principles to career decisions. Postgrad Med J.

[18] Majors. Winters JM, Wang H, Duwel LE, Spudich EA, Stanford JS (2018). Developing a backup plan: implementing a career-planning course for undergraduate biology. J Microbiol Biol Educ.

[19] Bansal A, Pusey J, Shah R, Tolley A (2023). Development and evaluation of an extra-curricular programme focussing on high impact career opportunities for medical professionals. PLoS One.

[20] Iwamuro M, Shiraha H, Okada H (2022). Gastric polyps' regression after potassium-competitive acid blocker cessation. J Gen Fam Med.

[21] McGaghie WC, Issenberg SB, Petrusa ER, Scalese RJ (2010). A critical review of simulation-based medical education research: 2003-2009. Med Educ.

[22] Komasawa N, Yokohira M (2023). Simulation-based education in the artificial intelligence era. Cureus.

[23] Johnson NR, Pelletier A, Chen X, Manning-Geist BL (2019). Learning in a high-stress clinical environment: stressors associated with medical students’ clerkship training on labor and delivery. Teach Learn Med.

[24] Lee A, Kuczmarska-Haas A, Dalwadi SM, Gillespie EF, Ludwig MS, Holliday EB, Chino F (2022). Family planning, fertility, and career decisions among female oncologists. JAMA Netw Open.

[25] Shimamura Y, Masuda K, Anbo Y, Sugaya F, Furuta Y (2022). A single-center comparative analysis of outpatients with and without COVID-19 in Sapporo, Japan. J Gen Fam Med.

[26] (2024). The Model Core Curriculum Expert Research Committee: The Model Core Curriculum for Medical Education in Japan. https://www.mext.go.jp/content/20230315-mxt_igaku-000026049_00003.pdf.

[27] Perumalswami CR, Takenoshita S, Tanabe A (2020). Workplace resources, mentorship, and burnout in early career physician-scientists: a cross sectional study in Japan. BMC Med Educ.

[28] Komasawa N, Terasaki F, Nakano T, Saura R, Kawata R (2020). A text mining analysis of perceptions of the COVID-19 pandemic among final-year medical students. Acute Med Surg.

[29] Komasawa N, Berg BW (2016). Interprofessional simulation training for perioperative management team development and patient safety. J Perioper Pract.

[30] Komasawa N, Yokohira M (2023). Learner-centered experience-based medical education in an AI-driven society: a literature review. Cureus.

